# In Situ Water Quantification in Natural Deep Eutectic Solvents Using Portable Raman Spectroscopy

**DOI:** 10.3390/molecules26185488

**Published:** 2021-09-09

**Authors:** Suha Elderderi, Laura Wils, Charlotte Leman-Loubière, Hugh J. Byrne, Igor Chourpa, Cécile Enguehard-Gueiffier, Emilie Munnier, Abdalla A. Elbashir, Leslie Boudesocque-Delaye, Franck Bonnier

**Affiliations:** 1 EA 6295 Nanomédicaments et Nanosondes, Faculté de Pharmacie, Université de Tours, 31 Avenue Monge, 37200 Tours, France; suha.elderderimohmedabdelrhman@etu.univ-tours.fr (S.E.); igor.chourpa@univ-tours.fr (I.C.); emilie.munnier@univ-tours.fr (E.M.); 2 Department of Pharmaceutical Chemistry, Faculty of Pharmacy, University of Gezira, P.O. Box 20, Wad Madani 21111, Sudan; 3 EA 7502 Synthèse et Isolement de Molécules BioActives (SIMBA), Université de Tours, 31 Avenue Monge, 37200 Tours, France; laura.wils@etu.univ-tours.fr (L.W.); charlotte.leman-loubiere@clarins.com (C.L.-L.); cecile.enguehard-gueiffier@univ-tours.fr (C.E.-G.); leslie.boudesocque@univ-tours.fr (L.B.-D.); 4 FOCAS Research Institute, TU Dublin-City Campus, Dublin 8, Ireland; hugh.byrne@tudublin.ie; 5 Department of Chemistry, Faculty of Science, University of Khartoum, P.O. Box 321, Khartoum 11115, Sudan; bashir_gezira@yahoo.com; 6 Department of Chemistry, College of Science, King Faisal University, P.O. Box 400, Al-Ahsa 31982, Saudi Arabia

**Keywords:** deep eutectic solvent, portable Raman spectroscopy, label free water quantification, in situ analysis, partial least squares regression

## Abstract

Raman spectroscopy is a label-free, non-destructive, non-invasive analytical tool that provides insight into the molecular composition of samples with minimum or no sample preparation. The increased availability of commercial portable Raman devices presents a potentially easy and convenient analytical solution for day-to-day analysis in laboratories and production lines. However, their performance for highly specific and sensitive analysis applications has not been extensively evaluated. This study performs a direct comparison of such a commercially available, portable Raman system, with a research grade Raman microscope system for the analysis of water content of Natural Deep Eutectic Solvents (NADES). NADES are renewable, biodegradable and easily tunable “green” solvents, outcompeting existing organic solvents for applications in extraction from biomass, biocatalysis, and nanoparticle synthesis. Water content in NADES is, however, a critical parameter, affecting their properties, optimal use and extraction efficiency. In the present study, portable Raman spectroscopy coupled with Partial Least Squares Regression (PLSR) is investigated for rapid determination of water content in NADES samples in situ, i.e., directly in glassware. Three NADES systems, namely Betaine Glycerol (BG), Choline Chloride Glycerol (CCG) and Glucose Glycerol (GG), containing a range of water concentrations between 0% (*w*/*w*) and 28.5% (*w*/*w*), were studied. The results are directly compared with previously published studies of the same systems, using a research grade Raman microscope. PLSR results demonstrate the reliability of the analysis, surrendering R^2^ values above 0.99. Root Mean Square Errors Prediction (RMSEP) of 0.6805%, 0.9859% and 1.2907% *w*/*w* were found for respectively unknown CCG, BG and GG samples using the portable device compared to 0.4715%, 0.3437% and 0.7409% *w*/*w* previously obtained by analysis in quartz cuvettes with a Raman confocal microscope. Despite the relatively higher values of RMSEP observed, the comparison of the percentage of relative errors in the predicted concentration highlights that, overall, the portable device delivers accuracy below 5%. Ultimately, it has been demonstrated that portable Raman spectroscopy enables accurate quantification of water in NADES directly through glass vials without the requirement for sample withdrawal. Such compact instruments provide solvent and consumable free analysis for rapid analysis directly in laboratories and for non-expert users. Portable Raman is a promising approach for high throughput monitoring of water content in NADES that can support the development of new analytical protocols in the field of green chemistry in research and development laboratories but also in the industry as a routine quality control tool.

## 1. Introduction

Raman spectroscopy is a cost-effective, label free and non-destructive analytical tool [1] that delivers specific molecular information to enable the determination of the chemical composition of samples within seconds, with minimum or no requirements for sample preparation. The technique is based on the illumination of samples with a monochromatic laser source, resulting in a fraction of the light being inelastically scattered (the Raman effect) which can be collected as spectral signatures [2]. Raman spectroscopy is considered as an approach well suited for the monitoring and control of chemical and pharmaceutical processes [3,4], end point prediction of chemical synthesis reactions [5] or monitoring of polymorphic transformation in crystallisation processes [6,7]. In the microscopic mode, Raman spectroscopy can access molecular information at the micron level [8,9], and notable applications have been extensively covered in the literature, e.g., the mapping of biological tissues and cells for diagnosis or drug interactions [10,11,12,13,14,15]. Raman systems designed for research are generally equipped with multiple laser sources and offer a multitude of customizable parameters to optimise the data collection, such as adjustable pinhole, high magnification objectives and interchangeable gratings for different spectral resolutions. However, numerous applications aiming at routine analysis in laboratories, or in situ analysis in industrial settings, do not require such performances and sophisticated equipment. As an example, Raman spectra can be recorded from a sample directly in its container [16,17,18]. The analysis can be performed directly through packaging materials for identification of raw pharmaceutical materials in amber glass bottles, opaque polypropylene containers, paper, blue plastic sacking [16] and USP vials [17], enabling, for example, characterisation of artesunate antimalarial drugs through blister bags [18] or discrimination and quantification of chemotherapeutic solutions in glass bottles [19] or perfusion plastic bags [20]. In this configuration, the signal is collected in situ (or non-invasively) from a voxel corresponding to a reduced volume into the sample without requirement for samples withdrawal. In recent years, technological achievements have greatly improved the transferability of Raman spectroscopy to a wider range of applications with miniaturisation of instruments [21].

Portable and handheld devices are considered as potential game-changers for field or bedside analysis, providing the performance of highly specified laboratory instruments without the requirement for a dedicated analytical space with bulky and expensive equipment. Recent publications report a number of applications for food industry [22,23], forensic science [24], archaeological science [25], raw material identification [26] and counterfeit analysis for pharmaceuticals [27,28,29,30]. The quality of the data and the reproducibility of analysis open perspectives for accurate quantitative analysis, as demonstrated for antineoplastic solutions directly in glass vials [31] or in plastic bags [32], and active pharmaceutical ingredients directly in tablets [33] or in glass vials [34]. The new generation of commercial portable Raman devices represents a potentially easy and convenient analytical solution for day-to-day analysis in laboratories and industrial production line environments. However, it is important to demonstrate their performance for high accuracy analytical applications.

The industrial transition to green and sustainable production processes has led to intensive research into alternatives to organic solvent-based processing. In this context, Natural Deep Eutectic Solvents (NADES) have attracted the interest of researchers from a diverse range of different analytical and applications fields [35], biotechnology [36], energy [37], water remediation [38], food industry [39], cosmetics and pharmaceutics [40,41], etc. NADES are solvents of great interest in chemistry, due to their unique solvent properties, including high extraction ability for some natural products and high solubilisation strength of a wide range of organic and inorganic compounds [35,42,43,44]. NADES are simply prepared, cost effective, eco-friendly green solvents outcompeting existing organic solvents [45], in addition to being renewable and biodegradable [43] and easily tunable [46]. NADES applications have been reported for extraction of phenolic compounds for cosmetic, pharmaceutical and food industries [47,48,49,50], but they are also evaluated in a broad range of fields including analytical chemistry [35], organic synthesis [51], biotechnologies [52], electrochemistry [53], or nanotechnology [54,55,56]. NADES are viscous solvents [46], which is considered a major barrier in analytical chemistry applications [46,57,58,59]. Although the constituent compounds of NADES are often hygroscopic by nature, and hence, upon preparation samples contain an initial water content < 1% *w*/*w*, the controlled addition of water can be employed to systematically, for example, decrease the viscosity and improve solvation and mass transfer operations, therefore ensuring maximum efficiency during extraction [57,58,59,60]. Depending on the type of NADES, water molar concentration ratio is critical for certain applications, such as enzyme reactions, and dissolution of compounds which are major uses in cosmetics and pharmaceutical areas [46,58]. Moreover, the polarity of the NADES increases with water content and it strongly affects the solubilisation capacity of the NADES, depending on the nature of solutes [46]. Increased water content can weaken the interactions between the NADES and the target compounds, as well as the interactions between the components of the NADES, until complete disruption occurs [59]. The control of water content and the stability of NADES-formulated products remains one major bottleneck to an extensive industrial use of NADES [43,46,58]. Quantification of water content is therefore essential to ensure the reproducibility of experiments, especially after a storage period, because NADES tend to accumulate water from the ambient air, added for a specific application for optimal use, or from the biomass (plants, algae) during extraction [43,46,58].

Karel Fisher (KF) is the gold standard for quantification of residual water with numerous examples in organic solvents [61], plant extracts [62] or in food [63,64]. Despite the sensitivity of the method, the large volumes of reagent and solvent consumed for titration (especially for high water content) and the time requirements for analysis of large sample cohorts [65] motivate the development of alternatives. The gravimetric method is the simplest, solvent-free, cost-effective technique, which measures the weight loss in sample due to water evaporation after drying under heating. However, a lack of repeatability in results is observed due to thermal decomposition of sample or for volatile samples [65]. Hydrogen Nuclear Magnetic Resonance (NMR), Near Infrared spectroscopy (NIR) and Fourier Transformed Infrared Spectroscopy (FTIR) have demonstrated potential for water quantification [66,67,68,69,70]. Recently, Elderderi et al. [71,72] have reported studies highlighting the application of Attenuated Total Reflectance Infrared spectroscopy (ATR-IR) and Raman confocal microscopy, coupled to Partial Least Squares Regressions (PLSR), to estimate systematic variations in water concentration added to 3 NADES Betaine Glycerol (BG), Choline Chloride Glycerol (CCG) and Glucose glycerol (GG). However, all current methods suffer the same limitation, i.e., a fraction of the samples has to be withdrawn for analysis. Therefore, the present study aims to demonstrate how portable Raman instruments could be used for rapid determination of added water content in NADES samples in situ, i.e., directly in glassware, providing an accessible solvent, consumable free and cost-effective analytical tool for high throughput analysis. A standard addition protocol has been used to prepare a range of samples with controlled added concentration of water corresponding to industrial use for extraction purpose. The results are directly compared with previously published studies to evaluate the performances of the portable device tested against a research grade Raman microscope.

## 2. Materials and Methods

### 2.1. Reagents

Anhydrous betaine (Acros Organics^TM^, 98%, Geel, Belgium), choline chloride (Acros Organics ^TM^, 99%, Geel, Belgium), and α-d-(+)-glucose (Acros Organics ^TM^, >99% Geel, Belgium) and Glycerol (Fisher BioReagents™, >99%, Fisher Scientific, Illkirch, France) were purchased from Fisher Scientific SAS (Illkirch, France). Water was purified using a Milli-Q system (Millipore Corporation, Bedford, MA, USA).

### 2.2. Preparation of Natural Deep Eutectic Solvents (NADES)

NADES are a eutectic mixture of two or more compounds composed of a hydrogen bond acceptor (HBA) and a hydrogen bonding donor (HBD). In this study, three different hydrogen bond acceptors (choline chloride, betaine, and glucose) have been used with the same hydrogen bond donor (i.e., glycerol), in a molar ratio of 1:2, 1:8, and 1:3 for CCG, BG and GG NADES systems studied respectively. NADES were prepared using a heating method [58] by stirring the two components (HBA and HBD) and heating the sample at 50 °C for CCG and BG and 80 °C for GG until a homogeneous colourless phase was formed [58]. The compounds used are hygroscopic by nature, and hence, upon preparation, samples contain an initial water content of <1% *w*/*w*. For the purpose of the study, the same stock for pure compounds were used, and all samples were prepared and analysed in short period of time to ensure the initial water content was consistent. Nine samples with systematically varied added water concentrations have been prepared for each NADES studied, using the standard addition protocol. During the preparation, known amounts of water were added to the mixture to yield a set of 9 samples, ranging from 0% to ~28.5% *w*/*w* added water concentration. Samples prepared for Raman analysis are listed in Table 1. All samples were prepared by weighing with an analytical balance of precision of 0.1 mg. For each concentration, a total mass of 5g of NADES was prepared, resulting in errors in references concentration provided in Table 1 in the order of 0.002%. For each sample, 3 sets were prepared. Spectral data collected from SET_01 and SET_02 are used as the training sets (calibration/validation) of the quantitative model, while SET_03 is used solely as an independent test, i.e., unknown samples (see Section 2.3.2).

### 2.3. Data Collection and Data Handling

#### 2.3.1. Data Collection

Raman spectra were collected using a portable Raman, Enspectr R532^®^ (EnSpectr, UK) instrument, of dimensions 222 × 145 × 55 mm and weight 1.5 kg. It is equipped with a 30 mW 532 nm laser source, the intensity of which was set to 15 mW at the sample to avoid photo damage. For the purpose of the study, the portable device was used with the sample holder provided by the manufacturer and designed for glass vials, enabling analysis of liquids. Presently, 2 mL of NADES were placed in transparent glass vials (Interchim, Montluçon, France). Spectra were collected over the range 160 to 4000 cm^−1^ with a spectral resolution of ~4–6 cm^−1^ (1800 lines/mm grating). Two accumulations of 10 s were acquired for each spectrum. For each NADES, 3 SETs of samples were independently prepared and analysed on different days. For each SET, the vial of a given concentration was positioned in the holder and 5 spectra were collected in a row, to assess the repeatability of measurements. Then, the sample was removed, and another concentration was tested. All concentrations of a given SET were analysed in a randomised fashion to avoid bias in interpretation. Once all vials were analysed, the entire operation was repeated 2 more times. Ultimately, all vials were analysed 3 times, resulting in 135 spectra per SET, 400 spectra per NADES and 1200 spectra for the whole study.

#### 2.3.2. Data Handling

Raman spectra were pre-processed and analysed using MATLAB^®^ (The Mathworks, Natick, MA, USA). Data were subjected to a Rubber band baseline correction followed by vector normalisation (RB-VN). Although the rubber band is an algorithm than can calculate a polynomial baseline to be subtracted from data [73,74], presently, the method was applied with a polynomial order set to 1, to avoid overcorrection of spectra [75]. The vector normalisation was applied, calculating the ratio of spectra to their respective Euclidian norms [2]. Pre-processed spectra were analysed with Partial Least Squares Regression (PLSR). PLSR is a widely used method to extract quantitative information from spectral data sets [76]. The multivariate method is applied simultaneously to the full spectral range, enabling identification of the relevant variables (wavenumbers) reflecting modifications in bands positions, intensities and shapes correlated with the systematic variation of water added to the NADES. PLSR is particularly powerful to construct linear models from spectra collected from mixtures displaying numerous partially overlapping peaks. The quality of the results and the reliability of the model were assessed using the Root Mean Square Error Cross Validation (RMSECV), the Root Mean Square Error Prediction (RMSEP), the linearity between the experimental and predicted concentrations (R^2^) and accuracy in predicted concentrations (relative error of the predictive concentration compared to the true value, expressed as %). For each NADES system analysed, 3 sets of samples have been prepared: SET_01 (n = 9), SET_02 (n = 9) and SET_03 (n = 9). SET_01 and SET_02 have been used as the training set and subdivided into the calibration set (2/3 of data) and validation set (remaining 1/3 of data) using a leave K-Out Cross Validation (LOKCV) approach. The combination being multiple, a 100-fold iterative protocol was applied to provide an overall estimation of the reliability of the calibration model (RMSECV and R^2^). Then, SET_03 was used in the predictive models as independent samples, i.e., previously unknown to the PLSR model, to be determined. As described in Section 2.2, the samples in SET_03 are also prepared by weighing and hence their concentration is known; these values are not used during the training of PLSR model, but rather at the later stage to assess the performance of the model based on the RMSEP and the accuracy of the prediction concentration expressed by the % relative error compared to the target (true) concentration. In this study, predicted concentrations, RMSECV and RMSEP are expressed as added % *w*/*w* water concentration

## 3. Results and Discussion

### 3.1. Characterisation of Spectral Variability in NADES

*(a)*  *Analysis through Glass*

**Figure 1:**
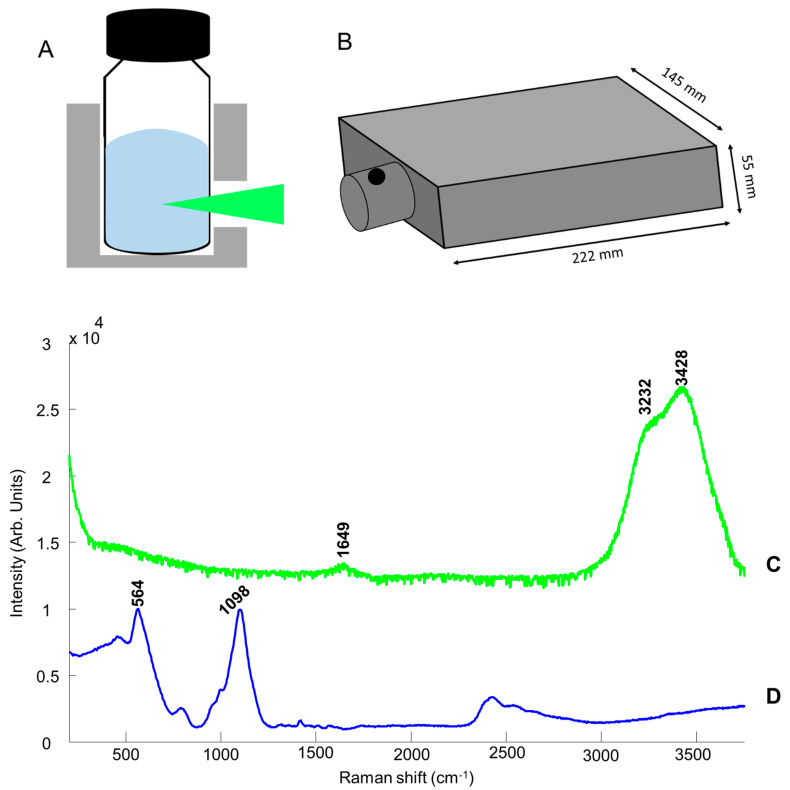
Illustration of the portable Raman device used: (**A**) sample holder, (**B**) the device, (**C**) mean Raman spectrum of the deionised water collected from a glass vial, and (**D**) a Raman spectrum of glass.

Figure 1A,B illustrates the experimental setup used to collect Raman spectra from NADES in glass vials. Although the Raman system can be handheld, for comparison to the system performance with the Raman grade instrument, the instrument was set on the benchtop, and the glass sample vial is placed into the sample holder. The 532 nm laser was focused inside the solution through the glass. Figure 1C presents a spectrum collected with the portable system from deionised water placed in one of the vials used for this study. H_2_O exhibits a weak feature at ~1649 cm^−1^ (scissoring bending) and broadband with two maxima at 3232 and 3428 cm^−1^, assigned to OH symmetric and asymmetric stretching modes of water molecules. Figure 1D shows a typical glass spectrum with 2 robust characteristic features observed at 564 and 1098 cm^−1^ [77,78]. These are absent in the spectrum of water recorded inside the vial, and therefore the solution inside the vials can be analysed without any contribution from glass features, thanks to the 75 mm focal length of the device, enabling focusing of the laser inside the solution.

*(b)*  *Choline Chloride Glycerol (CCG) NADES*

**Figure 2:**
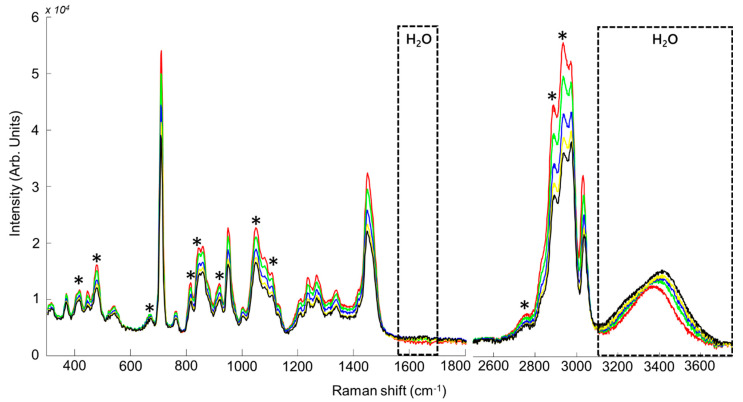
Mean Raman spectra for CCG samples. Concentrations for water are respectively 0% (red), 9.1% (green), 16.68% (blue), 23.08% (yellow), and 28.56% (black). * Indicates bands corresponding to glycerol. The dotted boxes indicate the positions of H_2_O features.

Figure 2 presents the Raman spectra collected from CCG/water samples. For clarity, only the datasets with added water concentrations of 0%, 9.1%, 16.68%, 23.08% and 28.56% *w*/*w* are shown. There is an inverse correlation between added concentration of water and concentrations of other NADES compounds. Therefore, choline chloride and glycerol bands intensities in the fingerprint (300–1500 cm^−1^) and high wavenumbers (2600–3050 cm^−1^) regions tend to decrease gradually as the water concentration increases. While the OH scissoring bending band from water at ~1649 cm^−1^ appears weak compared to the other bands assigned to NADES compounds, changes in the OH symmetric and asymmetric stretching modes in the 3050–3700 cm^−1^ region are more easily observed, thanks to their stronger intensity.

The fingerprint region exhibits numerous, sharper features. For CCG, a mixed spectral signature originating from glycerol and choline chloride is observed (Figure 2). Assignments were based on literature [79,80], while bands positions were confirmed from spectra collected from the pure compounds (data not shown). The bands at 415 cm^−1^, 478 cm^−1^ (CCO rocking), 670 cm^−1^ (CCC deformation), 815 cm^−1^ (CC stretching), 845 cm^−1^ (CC stretching), 917 cm^−1^ (CH_2_ rocking) and 1106 cm^−1^ (CO stretching) are assigned to glycerol [80]. The bands at 370 cm^−1^ (N(CH_3_)_4_ bending), 447 cm^−1^ (N(CH_3_)_4_ bending), 711 cm^−1^ (CH_2_ in-plane rocking and N–C symmetrical stretching), 762 cm^−1^ (CH_2_ in-plane rocking), 950 cm^−1^ (N–C asymmetric stretching), 1132 cm^−1^ (CH_2_ out of plane bending), 1268 cm^−1^ (CH_2_ out of plane bending, N–C asymmetric stretching) and 1337 cm^−1^ (CH_2_ out of plane bending) are assigned to choline chloride [79]. The bands at 1050 and 1450 cm^−1^ result from mixed features contribution from glycerol at 1055 cm^−1^ (CO stretching) and 1464 cm^−1^ (CH_2_ deformation) [80] and choline chloride at 1052 cm^−1^ (C–C stretching) and 1448 cm^−1^ (CH_2_ scissors and CH_3_ deformation vibrations) respectively [79].

The high wavenumber region (2500–3750 cm^−1^) provides information on vibrations from single bonds from light elements, presently C–H and O–H. The weak band at 2746 cm^−1^ (C–H stretching) is assigned to glycerol [80]. The band appearing as a shoulder in spectra at 2834 cm^−1^ (CHO stretching) from choline chloride [79]. The band at 2888 cm^−1^ (symmetric C–H stretching from CH_2_) is mainly due to glycerol contribution [80]. The band at 2936 cm^−1^ results from combined contribution of the feature at 2947 cm^−1^ (antisymmetric C–H stretching from CH_2_) and the feature at 2936 cm^−1^ (CH_2_O stretching) respectively observed in the pure spectra of glycerol and choline chloride (data not shown) [79,80]. The bands at 2973 cm^−1^ (C–H symmetric stretching) and at 3031 cm^−1^ (CH_3_ symmetric stretching, CH_2_ symmetric stretching) are specifically assigned to choline chloride [79]. In the range 3010–3650 cm^−1^, a substantial change in the absorption profile is witnessed in the presence of H_2_O in NADES samples, including a broadening of the band and the emergence of a shoulder between 3200–3400 cm^−1^. The broad band is derived from the combined contributions from symmetric and antisymmetric OH vibrations originating from glycerol, choline chloride and water. While the spectrum of the 0% added water sample displays a single symmetric band at 3387 cm^−1^, attributed to combined glycerol and choline O–H stretching vibrational modes [79], increasing the added water concentration leads to modifications in both its intensity and shape. The two features at ≈3200 and 3430 cm^−1^ attributed to the OH stretching of water (see Figure 1C), have increased intensities while the relative contributions from choline and glycerol decrease. The inversion for the peak corresponding to CH_2_ vibrational mode of glycerol (~2936 cm^−1^) indicates that the interspecies H-bonding with water primarily involves glycerol-OH, as opposed to choline-OH [81].

*(c)*  *Betaine Glycerol (BG) NADES*

**Figure 3:**
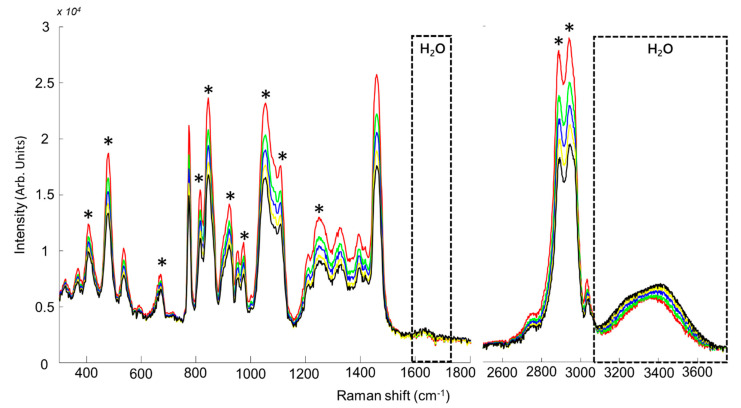
Mean Raman spectra for BG samples. Concentrations for water are respectively 0% (red), 9.085% (green), 16.655% (blue), 23.075% (yellow), and 28.56% (black). * Indicates bands corresponding to glycerol. The dotted boxes indicate the positions of H_2_O features.

Figure 3 shows the spectra of BG samples with 0%, 9.085%, 16.655%, 23.075% and 28.56% added water concentrations, as for the CCG system the opposite correlation between water and betaine-glycerol bands is observed. BG spectra are dominated with glycerol features with the most specific spectral ranges 400–500 cm^−1^, 800–900 cm^−1^ and 1000–1100 cm^−1^.

Contributions from betaine can be observed at 370 cm^−1^ (C-N (CH_3_) symmetric deformation) [57,82], 536 cm^−1^ (C=O bending), 774 cm^−1^ (C–N (CH_3_) symmetric stretch), 952 cm^−1^ (C–N (CH_3_) asymmetric stretch) [83], 1209 cm^−1^ (CO vibration), 1325 cm^−1^ (CCO–symmetric vibration) and 1395 cm^−1^ (CH_3_ scissoring bending) [83]. The band at 1460 cm^−1^ results from overlapping features of betaine at 1453 cm^−1^ (asymmetric stretching CH_3_) [83] and of glycerol at 1464 cm^−1^. In the high wavenumber region, similarly to CCG, the weak band at 2746 cm^−1^ and the 2 bands at 2888 and 2942 cm^−1^ are assigned to glycerol. The contributions from betaine are a shoulder at 2977 cm^−1^ (symmetric CH_2_ stretching) and the specific feature at 3035 cm^−1^ (asymmetric CH_3_ stretching) [83].

*(d)*  *Glucose Glycerol (GG) NADES*

**Figure 4:**
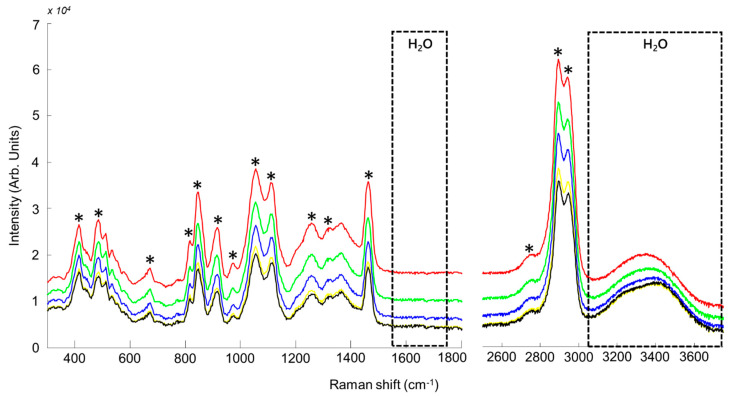
Mean Raman for GG samples (concentrations for water are respectively 0% (red), 9.094% (green), 16.67% (blue), 23.079% (yellow), and 28.57% (black). * Indicates bands corresponding to glycerol. The dotted boxes indicate the positions of H_2_O features.

Similarly, spectra of GG NADES samples with 0%, 9.094%, 16.67%, 23.079% and 28.57% added water concentration are shown in Figure 4. Again, the spectra are largely dominated by glycerol features. Contributions from glucose can be observed at 512 cm^−1^ (bending vibration CCO, C=O), 1111 cm^−1^ (CO stretching, CC stretching) and 1367 cm^−1^ (O-CH_2_ wagging) [80,84].

In the high wavenumbers region, although the pure spectrum of glucose displays two peaks, at 2901 cm^−1^ (C–H stretching) and 2953 cm^−1^ (C–H stretching of CH_2_) (data not shown) [84], these features are completely overlapped by the strong signature of glycerol.

### 3.2. Quantification of Spectral Variability in NADES (Training Sets)

**Figure 5:**
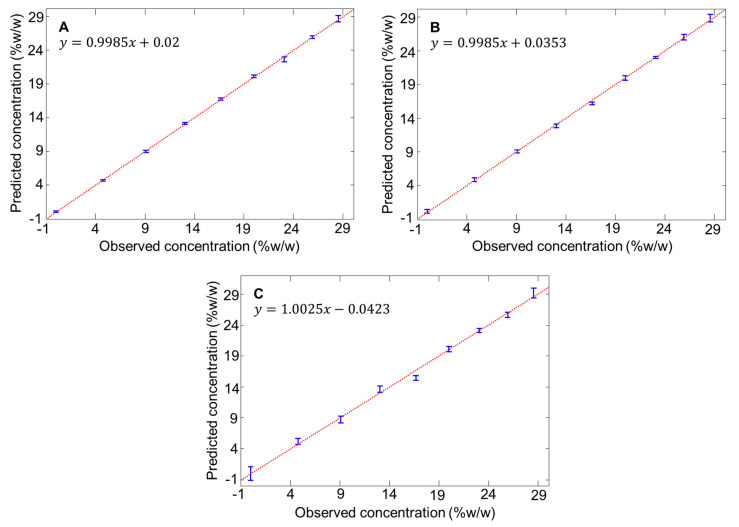
PLS regression models obtained from training sets using preprocessed spectra in the range 300–3750 cm^−1^ for CCG (**A**), BG (**B**) and GG (**C**).

In the fingerprint region, features of the NADES clearly dominate, while the water band is at best weakly observed. However, it appears that changes in the NADES compound concentrations by addition of water result in significant variations in band intensities. In the high wavenumber region, the presence of a broad band corresponding to OH symmetric and asymmetric stretching modes of water molecules with two maxima at 3232 cm^−1^ and 3428 cm^−1^ (Figure 2) suggests that the added water content could be more specifically followed in this spectral range. PLSR has been applied over the 300–3750 cm^−1^ spectral range to take into account both patterns observed in the construction of quantitative models. Figure 5 provides the regression plots obtained from the validation sets and Table 2 summarises the RMSECV (% *w*/*w*), R^2^ and number of latent variables used. For the 3 NADES, the R^2^ is above 0.99, while the RMSECV varies from 0.2728% *w*/*w* for CCG to 0.8157% *w*/*w* for GG. Overall, the results suggest a good linearity between the observed concentrations (reference added water concentrations) and the predicted added concentrations (estimated from Raman spectra).

**Figure 6:**
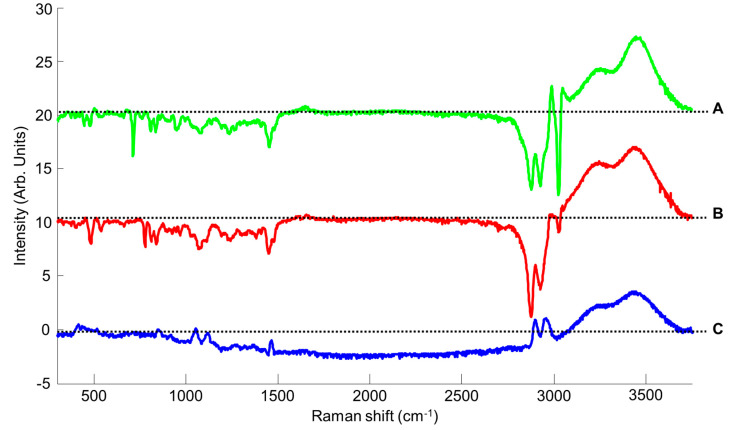
First regression coefficients from PLSR performed on CCG (**A**), BG (**B**) and GG (**C**) spectra. Dotted line indicates the zero baseline.

Regression coefficients highlight the positive contribution of water bands in the high wavenumber region for CCG, BG and GG NADES systems (Figure 6). This confirms that PLSR analysis uses changes in shapes (width, maximum intensity) occurring in the broad OH band due to the increase in added water concentration (see Section 3.1a) to construct the regression model. Spectral features from glycerol choline chloride, betaine and glucose contribute negatively to the regression coefficients. The intensities of these bands are also influenced systematically by the added water concentration, and therefore they play a key role in the construction of the quantitative model.

### 3.3. Estimating Added Water Concentration in Independent Samples (Test Sets)

For each NADES, a third set of 9 samples (SET_03), prepared and analysed independently, has been used as test samples in the PLSR models. The RMSEP value for CCG, BG and GG are 0.6805%, 0.9859% and 1.2907% *w*/*w*, respectively (Table 3). Similar to the training sets, the NADES GG display the lowest accuracy. To appreciate the outcome of the PLSR analysis, the mean estimated concentrations are gathered in Table 4. For each sample, the relative error % between the true concentration (prepared by weighing) and the predicted concentrations (estimated from spectra) is used to evaluate the accuracy of the models. Water concentrations in SET_03 are determined independently as unknown samples in the PLSR models. Overall, no aberrant values are observed, i.e., high unexplained random or systematic errors in predicted concentrations. This indicates that there is no interferences in results originating from poor reproducibility in data collection trough glass or in the standard addition protocol used to prepare samples.

For CCG, all unknown samples are determined with less than 5% relative error. For BG, relative errors are below 5% except for sample 2 with 34.039% corresponding to a predicted added water concentration of 6.375% *w*/*w*, instead of 4.756% *w*/*w*. It is a difference of 1.619% *w*/*w* compared to the true concentration. For GG, three samples gave relative errors above 5%, sample 2 (34.511%), sample 4 (13.055%) and sample 5 (6.191%) corresponding respectively to predicted concentrations of 6.409%, 11.345% and 15.639% *w*/*w* instead of the true concentrations 4.765%, 13.048% and 16.672% *w*/*w*. These are differences of 1.644% *w*/*w*, 1.703% *w*/*w* and 1.033% *w*/*w* compared to prepared added water concentrations. It is observed that the two highest relative errors are found for, respectively, sample 2 of both BG and GG NADES, with added water concentrations ≈ 4.7% *w*/*w*, the second lowest added water concentration in the range analysed, which extends up to ≈ 28.5% *w*/*w*. The observed errors can reflect a lack of sensitivity of portable Raman spectroscopy for NADES samples containing small amounts of water. For GG samples, the R^2^ = 0.9873 obtained with the test set (Table 3) suggests a slight reduction of linearity in the predictive model, which is confirmed by the RMSEP = 1.2907% *w*/*w* (Table 3) and the PLS regression model in Figure 5C.

### 3.4. General Discussion

The reported results clearly demonstrate the possibility to perform Raman analysis in situ without contribution from the glass (Figure 1, Figure 2, Figure 3 and Figure 4). In the literature, correction methods have been explored to remove the interference from substrates like glass in spectra [77,85]. However, avoiding undesirable contribution from the vial itself at the stage of data collection significantly simplifies subsequent preprocessing and data analysis steps. Presently, it is demonstrated that the spectral resolution ~4–6 cm^−1^ delivered by the portable system tested is perfectly suited to record Raman spectra encompassing enough information about the NADES composition to construct accurate quantitative models by means of PLSR. In terms of performances, the results can be compared to a recent study by Elderderi et al., reporting water quantification in NADES using a confocal Raman spectrometer, implying sample withdrawal and analysis using a quartz cuvette [72]. The RMSEP values with the Raman microscope were respectively 0.4715%, 0.3437% and 0.7409% *w*/*w* for CCG, BG and GG compared to 0.6805%, 0.9859% and 1.2907% *w*/*w*, presently obtained with the portable Raman directly into glass vials. While direct comparison of the results is to be done with caution, due to the use of different laser wavelength in the 2 studies, it is observed that in situ analysis in glass vials leads to an increase in RMSEP. Nevertheless, the relative errors calculated from the samples analysed as unknown (test sets) highlight that the portable Raman, similarly to the Raman microscope, enables estimation of the added water concentration in NADES below 5% error for most of samples. The main difference lies in samples n°2 of BG and GG with respectively 34.039% and 34.511% relative errors. For these 2 samples, the true concentrations (prepared by weighting) were respectively 4.756% and 4.765% *w*/*w* while the predicted concentrations are 6.375% and 6.409% *w*/*w*, an error of ~1.6% *w*/*w* added water concentration. There are no official guidelines specifying the threshold for acceptable errors in water quantification in NADES. However, one can consider that it would be system and application dependent. For instance, CCG, BG and GG NADES are commonly used with water content between 15% and 25% *w*/*w* for extraction purposes (presently corresponding to samples 5–7) [46]. Therefore, errors of roughly 1–2% *w*/*w* in the estimated added water concentrations for samples containing less than 5% *w*/*w* water using portable Raman analysis may not be relevant.

Portable Raman devices have great potential to enable rapid and withdrawal free monitoring of water in NADES. Performing the analysis directly in glassware can significantly accelerate the workflow and support high throughput monitoring, while there is no requirement for consumables. Multivariate approaches like PLSR are increasingly included in manufacturer software for automated data preprocessing and analysis, making the technique accessible. While no official guidelines and acceptance criteria are clearly established for water quantification in NADES, the repeatability and reproducibility of measurements achieved in situ are highly encouraging. Moreover, Raman spectroscopy is not only a rapid and reliable tool, as it also provides label free and solvent free analysis and the possibility to foresee protocols free of single use consumables to reduce waste to a minimum. Considering the high demands in green chemistry and for eco-friendly solvents, Raman spectroscopy has a key role to play to provide an analytical technique in harmony with the overall concept.

## 4. Conclusions

Portable Raman devices represents an easy and convenient analytical solution for in situ analysis in laboratories. It is a suitable technique for solutions analysis without any sample preparation, but more importantly non-invasively directly in glassware. In the current study, rapid and accurate quantification of added water was achieved in unknown samples for choline chloride glycerol, betaine glycerol and glucose glycerol NADES with RMSEP of 0.6805%, 0.9859% and 1.2907% *w*/*w* in added water concentrations.

Moreover, mean relative errors in predicted added water concentrations of 2.686%, 6.6115% and 8.10763% *w*/*w* were achieved, further supporting the technique reliability. Nowadays, NADES are described as green solvents in chemistry for plant extraction, which demands suitable analytical tools for efficient implementation and maximised economical and health benefits. This study highlights the potential of portable Raman spectroscopy to support the establishment of green chemistry protocols in the cosmetic and pharmaceutical fields, helping to optimise processes of extraction and purification of active molecules.

## Figures and Tables

**Table 1:** List of samples prepared for the 3 NADES studied.

Additional Water Concentration (% *w*/*w*)									
Sample n°	Betaine–Glycerol (BG)			Choline Chloride–Glycerol (CCG)			Glucose–Glycerol (GG)		
	SET_01	SET_02	SET_03	SET_01	SET_02	SET_03	SET_01	SET_02	SET_03
1	0.000	0.000	0.000	0.000	0.000	0.000	0.000	0.000	0.000
2	4.763	4.767	4.756	4.761	4.767	4.764	4.761	4.764	4.765
3	9.088	9.082	9.086	9.100	9.095	9.107	9.096	9.091	9.095
4	13.039	13.032	13.030	13.045	13.038	13.039	13.048	13.044	13.0486
5	16.649	16.656	16.660	16.664	16.685	16.681	16.667	16.677	16.672
6	19.976	20.007	20.003	19.999	19.995	20.006	20.005	20.006	20.007
7	23.066	23.075	23.085	23.095	23.080	23.079	23.0770	23.0772	23.085
8	25.926	25.910	25.911	25.935	25.920	25.923	25.935	25.930	25.940
9	28.592	28.548	28.551	28.540	28.559	28.588	28.577	28.564	28.580

**Table 2:** Summary of RMSECV obtained from PLSR applied on training SETs (SET_01 and SET_02).

	RMSECV (% *w*/*w* Added Water Concentration)	R^2^	LV
CCG	0.2728	0.9991	7
BG	0.3724	0.9985	6
GG	0.8157	0.9928	6

**Table 3:** Summary of RMSEP obtained from PLSR applied on Test SET (SET_03).

	RMSEP (% *w*/*w* Added Water Concentration)	R^2^
CCG	0.6805	0.9995
BG	0.9859	0.9956
GG	1.2907	0.9873

**Table 4:** Summary of accuracy for added water quantification in test samples (SET_03).

Relative Error (RE)%			
Sample n°	CCG	BG	GG
1	NA	NA	NA
2	4.025	**34.039**	**34.511**
3	1.358	4.749	2.436
4	0.376	4.460	**13.055**
5	1.122	3.357	**6.191**
6	2.692	2.114	2.531
7	3.291	2.518	1.698
8	4.287	0.009	1.325
9	4.337	1.646	3.114
Mean	2.686	6.612	8.108

## Data Availability

The data presented in this study are available on request from the corresponding author.

## Sample Availability

Samples of the compounds are not available from the authors.
